# Aesthetic and Functional Outcomes After Superficial Parotidectomy – Comparison of Three Reconstruction Techniques: An Interventional Clinical Study

**DOI:** 10.1055/s-0045-1809930

**Published:** 2025-10-16

**Authors:** Sherif M. Askar, Abd El-Raof Said, Tamer Oraby, Ibrahim Khaled, Mahmoud A. Megahed, Ali M. Awad

**Affiliations:** 1 Department of Otorhinolaryngology–Head and Neck Surgery, Faculty of Human Medicine, Zagazig University, Zagazig City, Sharkia Governorate, Egypt

**Keywords:** superficial parotidectomy, sternomastoid muscle flap, fat graft

## Abstract

**Introduction:**

Preauricular defects is one of the main concerns after superficial parotidectomy. Plastic surgeons have described many filling techniques to overcome the problem.

**Objective:**

To discuss and compare the esthetic and functional outcomes of three postsuperficial parotidectomy reconstruction techniques: partial-thickness, superiorly based sternocleidomastoid muscle flap, en-bloc fat grafting, and platelet-rich fibrin gel.

**Methods:**

We included 29 adult patients submitted to reconstruction after superficial parotidectomy by partial-thickness, superiorly based sternocleidomastoid muscle flap, en-bloc fat grafting, and platelet-rich fibrin gel. A subjective evaluation of the overall facial appearance was conducted through a 5-point visual analog scale filled out by the patient, a close relative, and 3 blind staff members.

**Results:**

Regarding the visual analog scale, in the comparison between the three groups (at the sixth and the twelfth months), the fat group reported the highest mean for satisfaction (3.4 ± 1.1 and 3.83 ± 0.97 respectively) and showed a highly-significant difference when compared to the sternocleidomastoid muscle flap and the platelet-rich fibrin gel groups ( *p* = 0.0001 and 0.016 respectively).

**Conclusion:**

Parotidectomy with immediate reconstruction of the surgical defect with an en-block fat graft provides better esthetic outcomes than sternocleidomastoid muscle flap and platelet-rich fibrin gel after 1 year. The sternocleidomastoid muscle flap and fat techniques resulted in minimal surgical-site morbidity and lower likelihood of developing Frey's syndrome. The fat graft yielded the best degree of cosmetic satisfaction with minimal morbidity. Fat overcorrection is recommended.

**Level of Evidence** : 4

## Introduction

The most common indication for superficial parotidectomy (SP) is the removal of a benign neoplasm; 75 to 80% of neoplasms of the parotid gland are benign. Pleomorphic adenoma and Warthin's tumor account for most of the tumors encountered. 1 The main target of SP is the removal of the entire benign tumor (with its capsule) without injuring the facial nerve. Surgical hazards include Frey's syndrome (FS) and numbness of the ear lobule. Several annoying esthetic issues might follow SP; facial asymmetry, scar deformity, and hollowed-out preauricular defects are the main concerns. Modern surgeons tend to deal with these ordeals through modifications in the classic incision, local flaps, and volumetric autogenous soft-tissue transfer to fill the surgical defect. 2 3 4 5 6 7 A review of the available English literature indicates that no single technique has gained universal agreement among surgeons so far. Moreover, the optimum protocol is still controversial.

Partial-thickness, superiorly based sternocleidomastoid muscle flap (SCF), which presents low donor-site morbidity, has its role among the various methods described to lessen the postparotidectomy defect. 4 5 8 On the other hand, it has been reported 9 10 that fat grafts yield primary satisfactory esthetic outcomes. Platelet-derived growth factors can stimulate tissue repair, support cellular proliferation and differentiation, and influence extracellular matrix deposition. Thus, platelet-rich fibrin gel (PRF) has been used in different surgical indications aiming at improving outcomes. 11 12 13 14 15 16 17 18

The goal of the present work was to study and compare the esthetic and functional outcomes of the three popular post-SP reconstruction techniques: SCF, en-bloc fat grafting, and PRF.

## Methods

### Settings

The current prospective, randomized, interventional clinical study was conducted at the Department of Otorhinolaryngology–Head and Neck Surgery of our institution from May 2018 to March 2023.

### Ethical Considerations

The institutional review board approved the research methodology (under number Zag IRB-1271). Informed written consent was obtained from the participants.

### Reporting Guidelines

Specific guidelines about the esthetic outcomes after parotidectomy are lacking. We followed the Clinical Practice Guideline of the American Society of Clinical Oncology (ASCO). 19

### Inclusion and Exclusion Criteria

We included adult patients submitted to SP for benign parotid lesions. Patients with malignant pathology, recurrent parotid lesions, and previous radiotherapy were excluded. The follow-up ranged from 28 to 70 (mean= 45 ± 3.28) months.

### Methods

After the preoperative preparation, the patients underwent SP with immediate reconstruction. A modified Blair's incision was used in all patients. The study group was randomly stratified into three subgroups according to the reconstruction technique: group 1–SCF; group 2–fat transfer; and group 3–PRF. The operative time was defined as the time to complete the reconstruction method; the time required for the SP and parotidectomy wound closure was not considered.

#### Surgical Technique for SP

The patient was placed as usual for SP, and the incision began as a standard preauricular curvilinear incision in front of the tragus, moving downwards around the inferior border of the lobule, the tip of the mastoid process, and then was continued forwards in the skin crease. The skin flaps were elevated, and then the greater auricular nerve was identified and dissected. The facial nerve trunk was identified by the standard anatomic landmarks. 1 2 The procedure was accomplished with the dissection and preservation of facial nerve branches from underlying parotid tissue.

**A) Assessment of the specimen volume**

The resected tissues were submerged in distilled water in a scaled specimen cup, and the overflowing volume of the water was defined as the specimen's volume (SV).

**B) SCF**

**Fig. 1:**
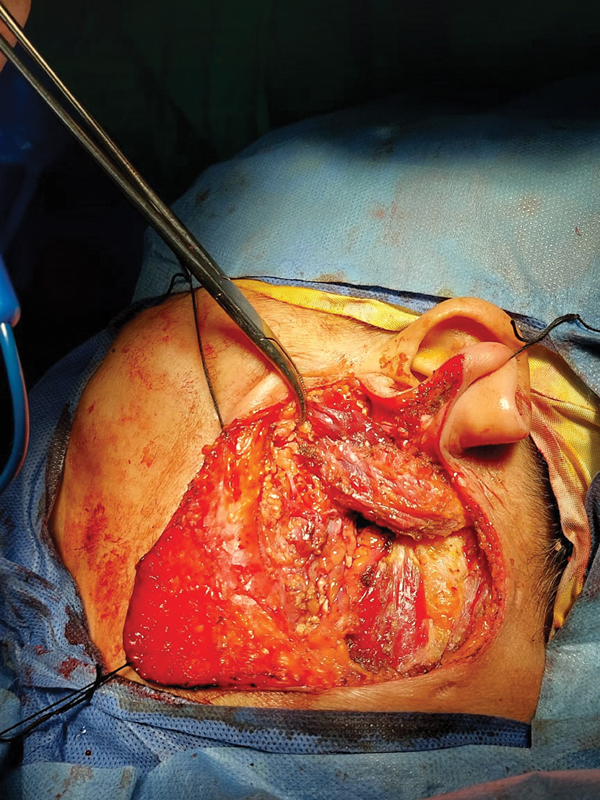
Partial-thickness, superiorly based sternocleidomastoid muscle flap.

The length of the SCF was first estimated by measuring the length of the defect; the thickness of the flap varied according to the size of the gap. The flap was elevated and rotated anteriorly to be sutured to the parotid fascia. This would cover the facial nerve (and its branches), the retromandibular vein, and the external carotid artery (and its terminal branches), and would fill the surgical defect. Then a drain was placed deep to the flap ( Fig. 1 ).

#### Fat Transfer Technique: (En-bloc Fat Technique)

**Fig. 2:**
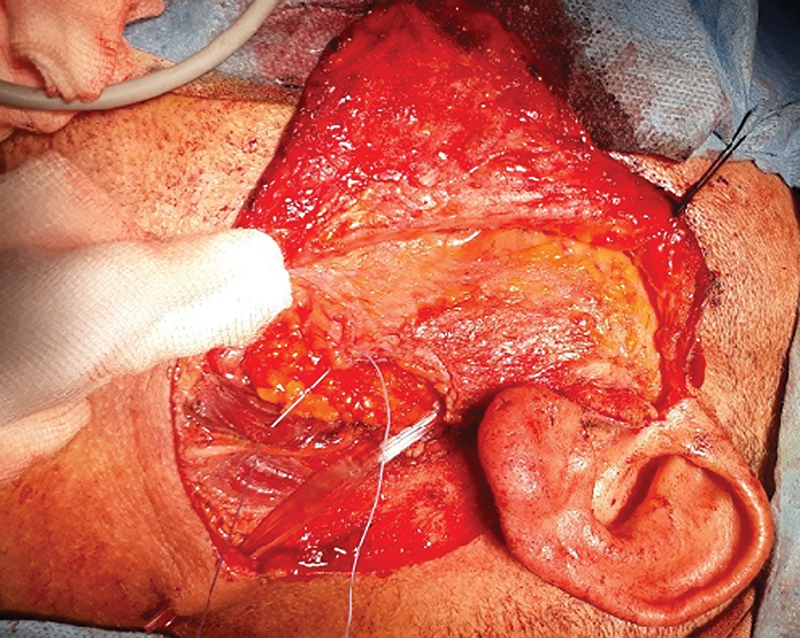
The fat graft in place.

A periumbilical incision was performed. The fat graft volume was assessed using the submerging technique in a scaled cup. The (en-bloc) fat graft was placed in the surgical defect and then sutured to the remaining parotid tissue (with Vicryl 3/0 sutures; Ethicon Inc.) to ensure graft fixation. The operative time for harvesting the fat graft and closure of the abdominal incision was calculated. Blood loss from the donor site was recorded. Non-suction rubber drains were used ( Fig. 2 ).

#### PRF

**Fig. 3:**
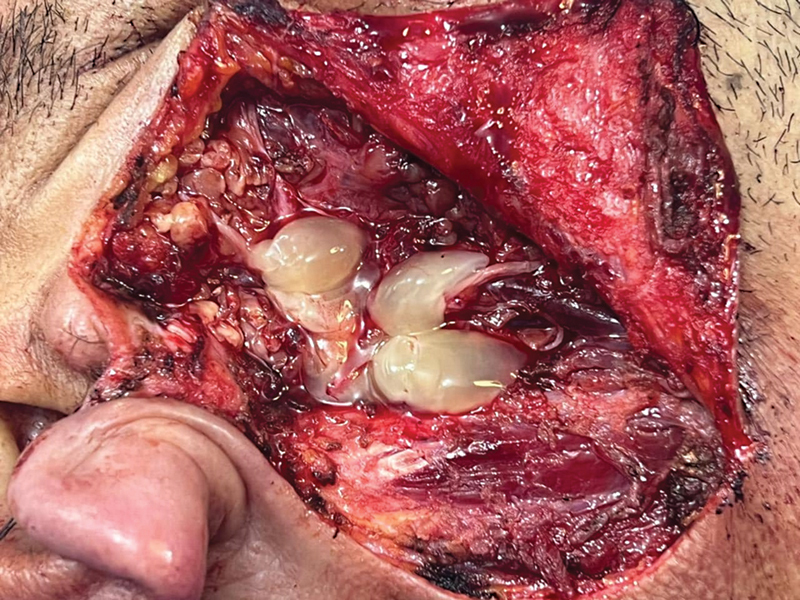
Platelet-rich fibrin gel in place.

For PRF preparation, a peripheral venous blood sample was drawn from the patient and then placed in 10-mL sterile tubes (with neither anticoagulants nor calcium). The tubes were centrifuged (Model 800 centrifuge, Jiangsu Zhengji Instruments Co., Ltd) for 10 minutes at 3,200 rpm. The blood would show three layers: the bottom layer (of red blood cells), the middle layer (of PRF, platelets, and white blood cells), and the top layer (of platelet-poor plasma). The PRF layer (50–60% of the blood in the tube) was extracted (30–40 cc) before application, and it was then placed on a sterile gauze pad to absorb the serum ( Fig. 3 ). 18 The fat graft and PRF volumes were estimated to be 40 to 50% greater than the SV. The wound was closed in layers over a non-suction drain.

#### Postoperative Follow-up

The parotid incision (and abdominal) drains were removed when a minimal collection was noticed. Examinations of the face and donor sites (abdomen) and functional outcomes were performed weekly for the first month, then monthly for 3 months, and then every 3 months until the end of the follow-up sessions. The functional evaluation included FS (starch iodine test 8 13 ), facial nerve integrity, and ear lobule sensation. The esthetic evaluation included facial deformity (preauricular/retromandibular defects) and the scar incision. A subjective evaluation of the overall facial appearance was accomplished through the visual analog scale–face (VAS-f; Table 1 ); this newly-designed 5-point scale was filled out by the individual patient, a close relative, and 3 blinded surgeons (5 = normal appearance, symmetrical to the opposite side; 4 = minimal asymmetry, barely noticeable from a short distance; 3 = mild asymmetry, noticeable but with no disfigurement; 2 = moderate asymmetry, mainly in the preauricular area; 1 = severe asymmetry, with deep preauricular/retromandibular groove (PRG); and 0 = severe asymmetry, with deep PRG and obvious scar). The VAS-f would provide a more objective evaluation of the results. On the calculation of results (collected from the 5 personnel), 0–10 were considered poor, 11–15, fair, 16–20, good, and > 20, as excellent.

**Table 1:** The designed visual analog scale–face (VAS-f)

Degree	Appearance
5	Normal appearance, symmetrical to the opposite side
4	Minimal asymmetry, barely noticeable from a short distance
3	Mild asymmetry, noticeable but with no disfigurement
2	Moderate asymmetry, mainly in the preauricular area
1	Severe asymmetry, with a deep preauricular/retromandibular groove
0	Severe asymmetry, with a deep preauricular/retromandibular groove and an obvious scar

### Statistical Analysis

All data were collected, tabulated, and statistically analyzed using the IBM SPSS Statistics for Windows (IBM Corp.) software, version 23.0. The quantitative data were expressed as the mean, standard deviation (SD), and range values, and the qualitative data, numbers and percentages. Analysis of variance (ANOVA) was used to compare more than two groups of normally distributed variables. The paired *t* -test was used to compare paired normally distributed variables. Values of *p* < 0.05 were considered statistically significant, and values < 0.001, highly significant.

## Results

### Patient Characteristics

We included 29 (17 female and 12 male) patients with a mean age of 50.48 ± 9.1 (range: (33–66) years, who were randomly (according to their numbers) distributed into 3 subgroups: Group 1 (SCF) included 11 (8 female and 3 male) patients, group 2 (fat), 9 (6 female and 3 male) patients, and group 3 (PRF), 9 (7 female and 2 male) patients. All of the subjects included unilateral benign parotid swelling for more than 6 months.

Among the sample, pleomorphic adenoma was the most common tumor mass encountered (19 patients; 65.52%). The tumor size ranged fro0m 14 to 30 cm ^3^ (mean: 21.4 ± 4.7) cm ^3^ ; the comparison of the groups regarding the size of the tumors was not significant ( *p* = 0.35).

### Outcome

Fat harvesting with wound closure was accomplished during the last steps of SP within 15 to 22 (mean: 18 ± 1.35) minutes. Blood loss from the periumbilical incision ranged from 10 to 20 (mean: 14.4 ± 4.6) mL. We did not report any case of postoperative fat necrosis (drainage of fat from the parotidectomy incision for a few days). The SCF was performed after finishing the SP; the time for flap harvesting and suturing ranged from 9 to 14 (mean: 11 ± 1.05) minutes. The comparison regarding fat harvesting and SCF times was significant (t = 12.7055; *p* = 0.0001).

Frey's syndrome was reported in 6 patients (20.69%): 3 from the fat group (33.3%), 2 from the PRF group (22.2%), and 1 from the SCF group (9.1%). By 12 months, 1 patient (from the PRF group) was still complaining of gustatory sweating.

Neck stiffness was reported in 2 male patients from the SCF group (18.2%). It improved in 3 to 6 months. Ear lobe numbness was reported by 6 patients (3 from the SCF, 1 from the fat, and 2 from the PRF group) which improved within 8 to 12 weeks. In the fat group, abdominal pain was reported in 2 patients (22.2%); the pain disappeared in 3 to 4 weeks. Abdominal seroma was reported in 1 patient (11.1%); conservative management was effective. At 6 months, no patients reported any problems regarding the abdominal incision. A comparison of the number of postoperative complications of the 3 groups did not show any significant results ( *p* = 0.407; Chio-squared [χ ^2^ ] = 1.7).

According to the VAS-f scores, in the sixth month, the SCF group reported fair results in 3 patients and good results in 8 patients. The fat group reported good results in 5 patients and excellent results in 4 patients. The PRF group reported fair results in all 9 patients. In the twelfth month, the SCF group reported good results in 9 patients and excellent results in 2 patients. The fat group reported good results in 2 patients and excellent results in 7 patients. And the PRF group reported fair results in 5 patients and good results in 4 patients.

**Fig. 4:**
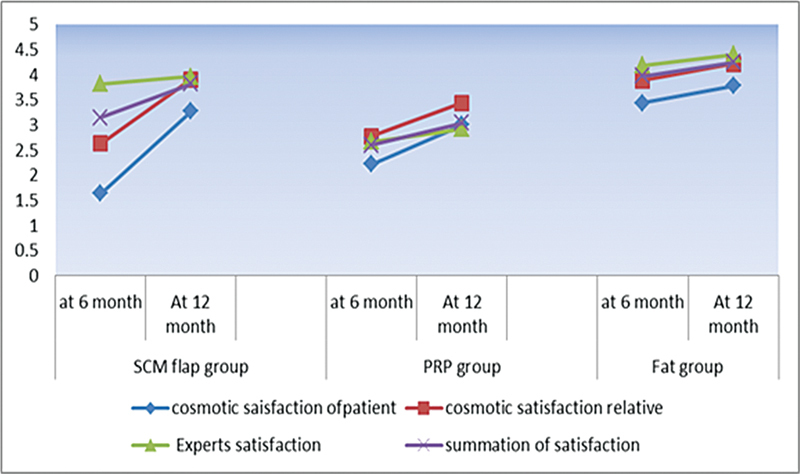
Mean scores on the visual analogue scale–face (VAS-f) at 6 and 12 months of three groups according to different evaluators.

Regarding the level of satisfaction, in the comparison of the 3 groups (at the sixth and twelfth months), the fat group reported the highest mean for satisfaction (3.4 ± 1.1 and 3.83 ± 0.97). The fat group showed a highly significant difference when compared to SCF and PRF groups ( *p* = 0.0001 and 0.016 respectively). The comparison of individual patient satisfaction at 6 and 12 months was highly significant in the SCF group (t = 5.2; *p* = 0.0001) and in the PRF group (t = 5.3; *p* = 0.001), while it was non-significant in the fat group (t = 1.2; *p* = 0.28). According to the opinion of the experts working in the study, the fat group reported the highest mean for satisfaction at the sixth (4.2 ± 0.41) and the twelfth months (4.4 ± 0.46). The comparison of the fat group with the PRF group revealed highly significant differences ( *p* = 0.0001). The comparison of the fat group with the SCF group revealed non-significant differences at the sixth month ( *p* = 0.068); the comparison of both groups revealed significant differences at the twelfth month ( *p* = 0.03). The comparison of the experts' opinions in the 6 and 12 months was significant in the fat group (t = 4; *p* = 0.001) and in the PRF group (t = 5.3; *p* = 0.001), while it was non-significant in the SCF group (t = 1.84; *p* = 0.096) ( Table 2 , Fig. 4 ).

**Table 2:** Mean postoperative scores of the sample (29 patients) on the visual analog scale–face (VAS-f) at 6 and 12 months

Variables	Study groups			f	*p*	Post-hoc test
	SCF	PRF	Fat transfer			
Cosmetic satisfaction according to the patients at 6 months	1.6 ± 1.1	2.2 ± 0.7	3.4 ± 1.1	8.1	0.002	(0.21)* (0.0001)** (.016)***
Cosmetic satisfaction according to the patients at 12 months	3.3 ± 0.9	3.0 ± 0.87	3.8 ± 0.97	1.7	0.205	–
Paired *t* -test; P1	5.2; 0.0001	5.3; 0.001	1.2; 0.28			
Cosmetic satisfaction according to the relatives at 6 months	2.6 ± 0.67	2.8 ± 0.7	3.9 ± 1.2	6.1	0.007	(0.72)* (0.003)** (0.01)***
Cosmetic satisfaction according to the relatives at 12 months	3.9 ± 0.54	3.4 ± 0.88	4.2 ± 1.2	1.7	0.196	–
Paired *t* -test; P1	6.5; 0.0001	4.0; 0.004	1.4; 0.19			
Experts score at 6 months	3.8 ± 0.37	2.7 ± 0.5	4.2 ± 0.41	31.1	0.0001	(0.0001)* (0.068)** 0.0001)***
Experts score 12 months	4 ± 0.35	2.9 ± 0.46	4.4 ± 0.46	29.2	0.0001	(0.0001)* (0.03)** (0.0001)***
Paired *t* -test; P1	1.84; 0.096	5.3; 0.001	4.0; 0.001			
Total score at 6 months	3.1 ± 0.14	2.6 ± 0.33	3.97 ± 0.35	46.2	0.0001	(0 001)* (0.0001)** 0.0001)***
Total score at 12 months	3.8 ± 0.34	3 ± 0.37	4.2 ± 0.31	28.5	0.0001	(0.0001)* (0.01)** (0.0001)***
Paired *t* -test; P1	6.3; 0.0001	10.0; 0.0001	4.0; 0.004			

**Abbreviations:** PRF: platelet-rich fibrin gel; SCF, partial-thickness, superiorly based sternocleidomastoid muscle flap.

**Notes:** P1: comparison within each group; *comparison between the SCF and PRF groups; **comparison between the SCF and fat transfer groups; ***comparison between the PRF and fat transfer groups; f, analysis of variance: *p* < 0.05: significant; *p* < 0.001: highly significant.

Regarding sex, the comparison among the whole sample groups after 6 and 12 months revealed higher levels of satisfaction among male subjects (in the three groups) than among female patients. This difference was significant in the fat and SCF groups ( *p* = 0.024 and 0.012 respectively in the sixth month) and was non-significant in the PRF group (in the sixth month, *p* = 0.051, and at the twelfth month, *p* = 0.064). The comparison of female and male participants in the same group showed significant differences in the SCF and in the fat groups ( *p* = 0.012 and 0.024 respectively) and showed non-significant results in the PRF group ( *p* = 0.051). Similar values were obtained in the twelfth month ( *p* = 0.017, 0.0059, and 0.064 respectively).

## Discussion

Facial nerve injury is the eminent complication of SP; however, the most common hazards affecting the individual patient's quality of life are the evident cosmetic defects, the defective sensation in the ear lobule, and FS. 2 3 4 5 Surgeons have presented different options to overcome the esthetic and the hollow defect problems. Besides the well-planned parotidectomy incision, these methods may employ a filler for the surgical defect, which may act as a biological barrier between the skin and the parotid bed to prevent FS; some researchers have tried SCF; other researchers, free fat grafts, while others still, PRF. Free flaps were discussed. 2 7 9 10 12 20

SCF had gained noticeable acceptance among surgeons. The flap is generous, easily harvested, it lies within the surgical field, and it presents a low risk of complications. It could be designed as superiorly- or inferiorly-based. 2 3 8 The immediate filling of the surgical defect with SCF yields good esthetic outcomes, with minimal donor site morbidity. 2 3 4 5

The PRF gel was recently used by different surgical specialties; it can be applied to fill cavities, as it contains several growth factors. Its basic components are fibrin matrix polymer, white blood cells, and blood aggregates. Moreover, it contains stem cells. Thus, it can promote tissue regeneration and provide adequate support for wound healing. Surgeons have discussed the application of PRF in SP and reported good outcomes. 11 12 13 14 15 21 22

Primary fat transfer after SP has yielded satisfactory esthetic outcomes. However, the technique requires a separate incision site and may result in complications at the donor site, such as hematoma, fat necrosis, and infection. The exact determination of the fat volume needed for reconstruction is another obstacle. 9 10 20 Tunca et al. 23 (2021) studied the fat graft resorption rates at 1 year and reported that a mean rate of 50.75 ± 21.20%. In the current study, we used the submerging technique for the measurement of the excised SV. At the sixth month, the abdominal incision presented minimal morbidity.

In the present study, FS was reported in 6 patients during the early follow-up visits. This number was improved to 2 patients by the twelfth month. The SCF group presented the best results regarding FS. Although the number is limited, these results might support SCF as a guard against FS. 24 25

In the current study, the authors employed a VAS-f scale for satisfaction. The fat group reported the highest means of satisfaction among patients, relatives, and experts in the 6 ^th^ and 12 ^th^ months. The lowest satisfaction was reported in the PRF group while the SCF group had a middle position among the groups. The comparison of the fat group with the other two groups showed highly significant differences at both visits. The comparison of the means of patients' satisfaction at 6 months and 12 months in the SCF group was highly significant, the PRF group reported significant results while a non-significant result was obtained in the fat group. Also, the results of this study showed more satisfaction among males than females.

### Limitations

The current study has limitations. First, the study was short-term and involved a relatively small number of patients. Second, the reporting of esthetic outcomes is hampered by their subjective nature and by interobserver variability. The authors tried to control this variability by reporting what was noticed by the patients themselves, a close relative, and three independent examiners. Third, there was a difference in volume replacement among the three reconstruction techniques, with fat grafting and PRF volumes being based on the SV; the SCF was dependent on surgical team estimation. Moreover, the current study did not enroll a control group. Thus, further prospective, long-term studies with a large sample are needed.

## Conclusion

Parotidectomy with immediate reconstruction of the surgical defect with an en-block fat graft provides better esthetic outcomes than SCF and PRF after 1 year. The SCF and fat techniques resulted in minimal surgical-site morbidity and less likelihood of developing FS. The fat graft yielded the best degree of cosmetic satisfaction, with minimal morbidity. Fat overcorrection is recommended.
